# An Algorithm for Idle-State Detection in Motor-Imagery-Based Brain-Computer Interface

**DOI:** 10.1155/2007/39714

**Published:** 2007-07-12

**Authors:** Dan Zhang, Yijun Wang, Xiaorong Gao, Bo Hong, Shangkai Gao

**Affiliations:** Department of Biomedical Engineering, School of Medicine, Tsinghua University, Beijing 100084, China

## Abstract

For a robust brain-computer interface (BCI) system based on motor imagery (MI), it should be able to tell when the subject is not concentrating on MI tasks (the “idle state”) so that real MI tasks could be extracted accurately. Moreover, because of the diversity of idle state, detecting idle state without training samples is as important as classifying MI tasks. In this paper, we propose an algorithm for solving this problem. A three-class classifier was constructed by combining two two-class classifiers, one specified for idle-state detection and the other for these two MI tasks. Common spatial subspace decomposition (CSSD) was used to extract the features of event-related desynchronization (ERD) in two motor imagery tasks. Then Fisher discriminant analysis (FDA) was employed in the design of two two-class classifiers for completion of detecting each task, respectively. The algorithm successfully provided a way to solve the problem of “idle-state detection without training samples.” The algorithm was applied to the dataset IVc from BCI competition III. A final result with mean square error of 0.30 was obtained on the testing set. This is the winning algorithm in BCI competition III. In addition, the algorithm was also validated by applying to the EEG data of an MI experiment including “idle” task.

## 1. INTRODUCTION

People who suffer from severe motor disabilities but are still cognitively intact, need an alternative method to interact with the environment. Over the past decades, the development of the technology called brain-computer interface (BCI) has provided a novel and promising communication channel for these patients. A BCI is a communication system in which messages or commands that an individual wishes to convey to the external world do not pass through the brain's normal motor output pathways [1]. A BCI system can “read out” the intention of the patients and translates it into physical commands which control devices that serve the patients.

There are various BCI systems using different methods to extract the subjects' intentions from their EEG signals. One of the practical BCI systems is based on motor imagery (MI) [2, 3]. The advantage of this type of BCI systems is that no external stimulation is needed. Current development of MI-based BCI is focused on how to discriminate different MI tasks and many algorithms could be applied to get satisfied results. However, during practical use of BCI system, users may stay free of MI tasks (i.e., “idle state” ) at all. In order to make the system robust, the BCI system should be able to effectively detect the “idle state” and act properly. Moreover, because idle state may refer to various brain activities except the specific MI tasks, so it is not possible to acquire representative training samples for classifier designing. Therefore, to develop a new algorithm which cannot only discriminate different MI tasks but also effectively detect the idle state without any training samples is critical for improving present MI-based BCI system.

In this paper, an algorithm which integrates two two-class classifiers with different parameters into one three-class classifier is proposed to overcome the difficulties mentioned above. The algorithm was applied to dataset IVc of BCI competition III. A final result with mean square error of 0.30 was obtained. In addition, an EEG experiment was carried out with similar setting as the one for the dataset of BCI competition III; the results showed the effectiveness of the proposed algorithm.

## 2. METHODOLOGY

### 2.1. Data description

#### 2.1.1. Dataset IVc of BCI competition III

BCI competitions are organized in order to foster the development of improved BCI technology by providing an unbiased validation of a variety of data-analysis techniques. The datasets of brain signals recorded during BCI experiments were from leading laboratories in BCI technology. Each data set is split into two parts: one part of labeled data (“training set”) and another part of unlabeled data (“test set”). Researchers worldwide could tune their methods to the training data and submit the output of their translation algorithms for the test data.

Dataset IVc of BCI competition III was recorded from one healthy subject. The training dataset consisted of 3 sessions (70 trials for each session). Visual cues (letter presentation) indicated for 3.5 seconds which of the following 2 motor imageries the subject should perform: *left hand*, *right foot*. The presentations of target cues were intermitted by periods of random length, 1.75 to 2.25 seconds, in which the subject could relax. The testing data (6 sessions, 70 trials each) was recorded more than 3 hours after the training data. The experimental setup was similar to the training sessions, but the motor imagery had to be performed for 1 second only, compared to 3.5 seconds in the training sessions. The intermitting periods ranged from 1.75 to 2.25 seconds as before. The other difference was that a new task *relax* was added (also with visual cues as indications). The subject was required not to perform any MI tasks during *relax* task. 118-channel EEG signals were recorded during the experiment with sampling rate of 1000 Hz (see [4] for more details).

Competitors of this data set were required to classify a set of single-trial electroencephalograph (EEG) data recorded from three-class tasks in the testing set. The output of the classification must be a real number between −1 and 1 (ideally, −1 for *left hand*, 0 for *relax*, and 1 for *right foot*). The challenge was that the training set consists of only two-class data (*left hand* and *right foot*). One problem existed for the classification is that the testing set contains a new class *relax* with no training data. And there are two other problems: (1) the MI tasks in the testing set were performed for only 1 second instead of 3.5 seconds as in the training set; (2) the testing data was recorded more than 3 hours after the training data was acquired, so the distribution of some EEG features could be effected by long-term nonstationarities. All of these are practical problems in current MI-based BCI systems. The main difficulty is detecting an additional state *relax* without training samples, which is the same as “idle state” we mentioned in the previous section.

#### 2.1.2. Datasets from our MI experiments

The data set provided by BCI competition III was acquired from only one subject and the details of the experiment were not so clear. In order to thoroughly investigate the effectiveness of our algorithm, an MI experiment was carried out with a similar paradigm.

Three right-handed volunteers (two females and one male, 22 to 24 years old) participated in this experiment. There were three kinds of tasks in the experiment: *left hand*, *right hand*, and *relax*. *left hand* and *right hand* referred to two MI tasks; while the subject was required not to perform any MI tasks during *relax* period. The subject was informed about which task to be performed by a visual cue on a PC screen before each trial. The trials lasted for 4 seconds with intermitting period of 2 seconds. 32-channel EEG (ActiveTwo system, BioSemi Instrumentation, Netherland) was recorded at the scalp over the motor cortex areas with a sampling rate of 256 Hz. For every subject, 50 trials for each task were collected.

Compared to the data set of BCI competition III, “*relax* with no training data” was emphasized while the other issues were ignored: the tasks were performed for 4 seconds instead of 3.5 seconds/1 second and all trials were performed continuously. The purpose of increasing trial time was to improve the performance because it was difficult to get satisfied results for normal subjects in such a short time as 1 second. And long-term nonstationarities were not concerned here for the complexities and characteristics of MI tasks.

### 2.2. Feature selection

Motor imagery can be seen as mental rehearsal of a motor act without any obvious motor output [2]. It is broadly accepted that mental imagination of movements involves similar EEG patterns that are also found in real movements. The main difference between real movements and MI is that execution would be blocked at some corticospinal level in the latter case [3]. Recent studies show that when performing motor imagination, mu (8–12 Hz) and beta (18–26 Hz) rhythms are found to reveal event-related synchronization and desynchronization (ERS/ERD) over sensorimotor cortex just like when one actually does the motor tasks [5].

**Figure 1:**
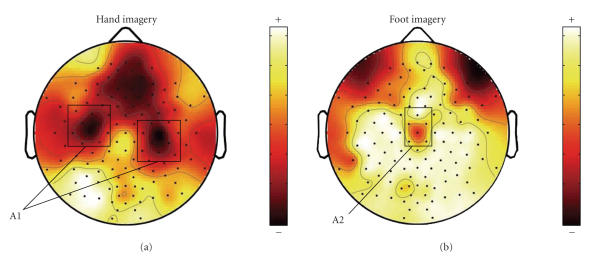
Averaged ERD spatial mappings of (a) *left hand* and (b) *right foot* in the training set.

Event-related desynchronization (ERD) represents power decrease in given frequency bands of the ongoing EEG activity [5]. Preparation of movement is typically accompanied by ERD in mu and beta rhythms over somatosensory or motor cortex. Figure 1 displays the averaged ERD spatial mappings of the two MI tasks in the training set. We use the ratio of power decrease in the imagery state and the power in the rest state as the quantification of ERD [5]. The brain regions containing significant ERD over motor cortex are marked as A1 and A2 in Figure 1. The ERD of *right-foot* imagery exists in the central area (A2) while the ERD of *left hand* is localized in both hemispheres (A1) with contralateral dominance. This difference is the basis for classifying *left-hand* and *right-foot* imageries.

The mental state of *relax* differs substantially from those of *left hand* and *right foot* since no brain activity patterns similar with MI is evoked. It is reasonable to assume that during a *relax* task there is no obvious ERD over somatosensory or motor cortex. So *relax* status can be distinguished from *left hand* and *right foot*. *left hand* can be recognized by existence of ERD in A1 area and *right-foot* is corresponding to the brain state with ERD in A2 area, while *relax* is just the brain state with no ERD in either A1 or A2 areas.

### 2.3. Feature extraction

The signals specific to the tasks are usually accompanied by interferences (such as noise, spontaneous EEG and other nontask activities). The common spatial subspace decomposition (CSSD) proposed by Wang et al. [6] was employed to extract the task-related source activities and to eliminate the background activities. The purpose of this method is to construct spatial filters which can distinguish two classes of signals based on simultaneous diagonalization of their covariance matrices [7].

In our method, we selected 37 EEG channels according to ERD distribution (see Figure 1), so only brain regions A1 and A2 are taken into consideration. Then we used the selected single-trial EEG data as the input matrix *X* with 37 (channels) by 280 (samples, 0.71–3.50 seconds) to construct spatial filters SF_*H*_ and SF_*F*_ for *left hand* and *right foot*, respectively. The spatial covariance of the EEG data can be obtained from

(1) $$C=X\cdot \;X^{T}$$.

The spatial covariance of each class is calculated as *C*_*H*_ and *C*_*F*_ by averaging over the trials in the corresponding class. The sum covariance matrix *C*_Sum_ is factorized into the product of eigenvectors and eigenvalues

(2) $$C_{\text{Sum}}=\;C_{F}+C_{H}=U_{0}\;\cdot \text{Σ}\cdot U_{0}^{T}$$.

The eigenvalues are assumed to be sorted in descending order. The whitening transformation matrix is then formed as

(3) $$P={\text{Σ}}^{-1/2}\cdot U_{0}^{T}$$.

If *C*_*H*_ and *C*_*F*_ are transformed as

(4) $$SC_{F}=P\cdot C_{F}\cdot P^{T},\;\;\;\;\;\;\;SC_{H}=P\cdot C_{H}\cdot P^{T}$$,

then *C*_*H*_ and *C*_*F*_ share common eigenvectors and the sum of the corresponding eigenvalues for the two matrices will always be 1, that is,

(5) $$SC_{F}=U\cdot {\text{Σ}}_{F}\cdot U^{T},\;\;\;\;SC_{H}=U\cdot {\text{Σ}}_{H}\cdot U^{T},\;\;\;\;{\text{Σ}}_{F}+{\text{Σ}}_{H}=I$$.

As the sum of two corresponding eigenvalues is always one, the eigenvector with largest eigenvalue for *S*_*F*_ has the smallest eigenvalue for *S*_*H*_. This transformation is an effective way for separating variances in the two matrices *C*_*H*_ and *C*_*F*_. Taking out the first *m*_*F*_ eigenvectors from *U* as *U*_*F*_ and the last *m*_*H*_ eigenvectors from *U* as *U*_*H*_, the spatial filters for class *F* and class *H* are

(6) $${\text{SF}}_{F}={\left(U_{F}\right)}^{T}\cdot P,\;\;\;\;\;\;\;\;{\text{SF}}_{H}={\left(U_{H}\;\right)}^{T}\cdot P$$.

The eigenvectors left in *U* correspond to the common spatial subspace of the two classes. The task-related components *S*_*H*_ and *S*_*F*_ are estimated by

(7) $$S_{F}={\text{SF}}_{F}\cdot X,\;\;\;\;\;\;S_{H}={\text{SF}}_{H}\cdot X$$.

*X* is a recorded data matrix of multichannel single-trial EEG. The columns of SF_*F*_^−1^/SF_*H*_^−1^ are spatial patterns corresponding to *right-foot*/*left-hand* components as time-invariant EEG source distribution vectors [8]. 

The features used for classification are obtained by decomposing the EEG using SF_*F*_ and SF_*H*_. The feature vectors of one single trial are defined as

(8) $$\begin{array}{c} f_{H,\;i}=\log\left(\mathrm{var}\left(S_{H,i}\right)\right),\;\;\;\;i=1,\ldots ,m_{H},\\ f_{F,i}=\log\left(\mathrm{var}\left(S_{F,i}\right)\right),\;\;\;\;i=1,\ldots ,m_{F}. \end{array}$$

*S*_*H*,*i*_/*S*_*F*,*i*_ represents the *i*th row vector of *S*_*H*_/*S*_*F*_. The log-transformation serves to approximate normal distribution of the data. Our experiences on the training set indicated that setting *m*_*F*_ = 3 and *m*_*H*_ = 3 was enough to get a fairly good performance.

**Figure 2:**
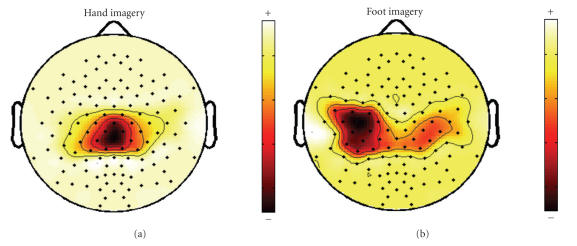
Most important spatial pattern of (a) *left hand* and (b) *right foot*.

During *left-hand* imagery, ERD occurs in region A1, leading to a relatively decreased EEG variance in this area. Therefore, *right foot* has a higher EEG variance than *left hand* in region A1. This behavior is reflected by large coefficients for channels covering region A1 in the spatial pattern corresponding to *right-foot* imagery. Figure 2 displays the most important spatial pattern of the two tasks. As shown in Figure 2(b), the most important spatial pattern of *right foot* accords with the ERD distribution of *left hand*. The spatial filter SF_*F*_ serves as extracting the component with a source distribution like the corresponding spatial pattern. Therefore, the component extracted by SF_*F*_ can be considered as the source activity concerning *left-hand* ERD, which has a significant distribution over region A1. A weak source activity leads to a small variance of relative scalp EEG, which is corresponding to significant ERD. Due to no ERD in region A1, the component of *right foot* has a larger variance than that of *left hand* when filtered by SF_*F*_, that is,

(9) $$\mathrm{var}\left({\text{SF}}_{F}\cdot X_{F}\right)>\mathrm{var}\left({\text{SF}}_{F}\cdot X_{H}\right)$$,

where *X*_*H*_ and *X*_*F*_ are single-trial EEG corresponding to *left hand* and *right foot*, respectively. We can also get another inequality as follows:

(10) $$\mathrm{var}\left({\text{SF}}_{H}\cdot X_{H}\right)>\mathrm{var}\left({\text{SF}}_{H}\cdot X_{F}\right)$$.

Note that according to the above definitions, *left-hand* MI causes a relatively increased EEG variance over area A2 (corresponding to *right-foot* task) because event-related desynchronization of EEG takes place on area A1. This behavior is reflected in large coefficients for electrodes on area A2 in the spatial filter of *left-hand* (SF_*H*_) [8], and vice versa for *right foot*. 

### 2.4. Classification method

The paper of Garrett et al. [9] showed that if features were properly extracted, the performance of linear classifiers can behave as well as that of complex nonlinear classifiers, so we simply used Fisher discriminant analysis (FDA) in our method. 

After using CSSD to extract ERD feature out of the training set, FDA was applied for classification and an accuracy of (99.1 ± 1.2)% was obtained on the training set using a 10 × 10-fold cross-validation. The result of FDA proves that there is no need to use other complicated methods.

### 2.5. Classification on the testing set

Denote *X*_*R*_ as a single-trial EEG of *relax*, as no ERD occurs in both regions A1 and A2 during *relax* tasks, the following inequalities come into existence:

(11) $$\mathrm{var}\left({\text{SF}}_{F}\cdot X_{R}\right)>\mathrm{var}\left({\text{SF}}_{F}\cdot X_{H}\right)$$,

(12) $$\mathrm{var}\left({\text{SF}}_{H}\cdot X_{R}\right)>\mathrm{var}\left({\text{SF}}_{H}\cdot X_{F}\right)$$.

Both components of *relax* and *right foot* are larger than that of *left hand* when filtered by SF_*F*_, so *left-hand* motor imageries can be discriminated from *right-foot*/*relax*. Similarly,* right foot* can be discriminated from *left-hand*/*relax* when filtered by SF_*H*_.

The required classification outputs of *left hand* and *right foot* are defined as −1 and +1. If we do a two-class classification based on the feature vectors *f*_*H*_ extracted by SF_*H*_ and set the classification outputs of *left hand* and *right foot* to −1 and +1 as required, then samples of *relax* are also classified to −1 as it is the same as *left hand* according to (9) and (11). Samples of *relax* are classified to +1 according to (10) and (12). Table 1 shows the different outputs of the three tasks in ideal conditions. Column “*f*_*F*_” and “*f*_*H*_” shows the two two-class classification results. Column “(*f*_*F*_ + *f*_*H*_)/2” represents the mean value of two outputs corresponding to *f*_*F*_ and *f*_*H*_ in the same row. Ideally, the two classifiers corresponding to “*f*_*F*_” and “*f*_*H*_” will result in opposite outputs for *relax* (+1/ −1 ) and the final classification result of *relax* can be set to 0 easily by “(*f*_*F*_ + *f*_*H*_)/2.” Therefore, it is possible to separate the three classes.

**Figure 3:**
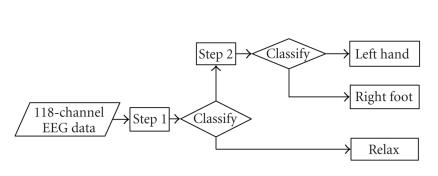
Flow chart of our algorithm.

Our strategy goes as following: at first, a two-class classifier was used to classify samples of *relax* to output 0 (see Table 1). Then the second two-class classifier was defined to classify the remaining samples into either *right foot* or *left hand*. The whole procedure of the classification algorithm is shown in Figure 3.

**Step 1 (Discriminating the relax trials).**

**Figure 4:**
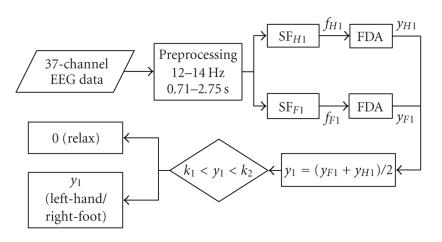
Classification process of Step 1.

The classification process of this step is showed in Figure 4. A subject-specific bandpass filter of 12–14 Hz (with most significant ERD feature for the subject of dataset IVc) and a time window of 0.71–3.50 seconds (eliminating the first 0.7 seconds as reaction time) were set for CSSD algorithm to calculate two spatial filters SF_*F*1_ and SF_*H*1_ from the training data. Because the duration of each trial in the testing set is much shorter than that of the training set, CSSD filter cannot get enough information with such a short time window to keep a high accuracy. Here we bring forward another assumption that the spatial pattern in the intermitted time (1.75–2.25 seconds) after a *relax* trial is similar to that of the *relax* trial (however, the intermitted time after an MI task cannot be simply considered as *relax* because the subject might keep on doing MI for a certain period even after he saw the cue for stop). With this assumption, a time window of 2.75 seconds (1 second for the task and at least 1.75 seconds for intermitted time) was selected as the input of the CSSD filters for the testing set. The effective duration of *relax* can thus be prolonged, making the classification results more reliable than those obtained by only using a short-time window.

*Bagging* strategy [10] was used here for reducing variance of classification accuracy. 160 trials were randomly selected out of all 210 trials in the training set to derive a classifier which was applied on each trial in the testing set. This process was repeated for 100 times, of which the classification outputs were averaged to get the final result. As shown in Figure 4 there are two FDA classifiers following two spatial filters SF_*F*1_ and SF_*H*1_. The outputs of these two classifiers (*y*_*F*1_ and *y*_*H*1_) were normalized to real number between −1 and 1 and were averaged to get a higher classification accuracy [11]. In Step 1 the averaging also has an effect of setting *relax* to 0.

After classification, two thresholds (upper boundary above 0 and lower boundary below 0) were determined manually, according to the distribution of training samples. The samples with classification outputs near 0 were labeled as *relax*. The remaining samples are left unlabeled to be fed as the input of Step 2. The process is shown as following where **k**_1_ and **k**_2_ denote the two thresholds:

(13) $$z=\{\begin{array}{ll} 0, & \text{if }k_{1}<y_{1}<k_{2},\\ y_{1},\; & \text{if }y_{1}<k_{1}\;\text{or}\;y_{1\;}>k_{2}. \end{array}$$

In our algorithm, we propose these two thresholds could be chosen to make *P*_1_ (in percentage) of the trials of MI tasks with nonzero classification output. (*P*_1_ was set to 70% for the results submitted to dataset IVc based on our former experiences)

**Step 2 (Discriminating the remaining trials).**

Step 1 is good for picking out *relax* but not optimal for classifying *left hand* and *right foot* because the intermitted time has been taken into consideration. During the intermitted time after *left hand* and *right foot*, the subject is told to “*relax*.” So a short time window (0.61–1.20 seconds) was defined as for this step. Besides, our investigation showed that a widepass band for temporal filtering (11–27 Hz) was better for classifying *left hand* and *right foot*. This wider frequency band including both mu and beta band is also good for generalization. The same time window as in Step 1 (0.71–3.50 seconds) was applied to calculate SF_*F*2_ and SF_*H*2_ with the training set.

**Figure 5:**
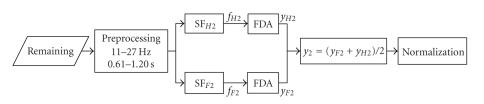
Classification process of Step 2.

The classification process of this step is shown in Figure 5. After classification we also set two thresholds manually to label samples with outputs congregating near −1 and 1 to *right hand* and *left foot*, respectively, and the others to a real number between −1 and 1. The normalization process is as follows, where *y* is the original output and *z* is the normalized output, *k*_3_ and *k*_4_ denote the two thresholds:

(14) $$\;z=\{\begin{array}{ll} -1, & \text{if }y_{2}<k_{3},\\ -\frac{y_{2}}{k_{3}}, & \text{if }k_{3}\leq y_{2}\leq 0,\\ \frac{y_{2}}{k_{4}}, & \text{if }0\leq y_{2}\leq k_{4},\\ 1, & \text{if }y_{2}>k_{4}. \end{array}$$

In our algorithm, we propose these two thresholds could be selected to make *P*_2_ (in percentage) of trials of MI tasks with classification outputs of ±1. (*P*_2_ was set to 70% for the results submitted to Data Set IVc).

For the data from our MI experiments, a time window of 0.5–4 seconds was applied to calculate spatial filters for both Steps 1 and 2. The frequency band used in Step 1 was subject-specific and 11–27 Hz were chosen in Step 2. Half samples of MI tasks (25 trials for *left hand*, 25 trials for *right hand*) were employed in the training set while the rest were used as the testing set. By randomly selecting trials for training, the classification process was repeated for 50 times to get average results. Features were extracted from both task and intermitting periods (6 seconds) in Step 1 while only task periods were considered in Step 2. Furthermore, we investigated how to choose threshold *k*_1_−*k*_4_ to get a better performance (refer to Section 3 for details).

## 3. RESULTS

The result of dataset IVc was evaluated by mean square error criterion. Defining the true labels for 420 trials in the testing set as *y*_1_, *y*_2_ ⋯ *y*_420_, and the classification outputs as *z*_1_, *z*_2_⋯ *z*_420_, the mean square error (MSE) was calculated as

(15) $$\text{MSE}=\frac{1}{420}{\sum_{i=1}^{420}\left(y_{i}-z_{i}\right)}^{2}\text{.}$$

**Figure 6:**
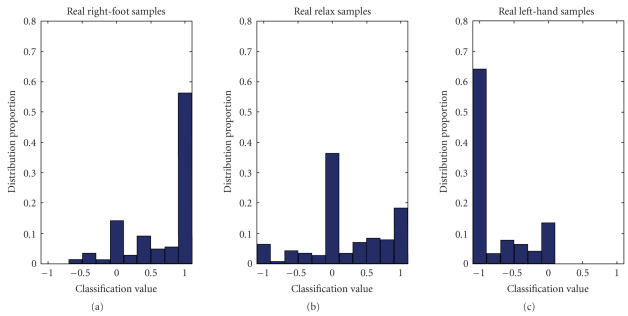
Distribution of classification results with respect to the three true labels.

As the winning algorithm in BCI competition III, a mean square error of 0.30 was achieved by our algorithm, which was much lower than the result of the second best competitor, who achieved 0.59 [12]. Figure 6 shows the distribution of the classification results of the three classes. Approximately 60% samples of true *left hand* and *right foot* are correctly classified to −1 and 1, and about 40% of *relax* samples are classified to 0. The particular strength of this method was that it managed to identify nearly half of the *relax* trials and none of the other submissions to this dataset handled the idle state well even if they discriminate the two MI tasks as well as our algorithm [12]. This could be the evidence that traditional algorithms are not so effective for classifying idle state. The results proved the effectiveness of this algorithm.

MSE is a specific performance measure used in BCI competition III. Two other measures with more direct meaning are defined as below.

(a) *Probability of detection (POD)*

For a certain task A, considering all trials of task A, let *N*_*D*_ denote the number of trials correctly detected as task A, *N*_*M*_ the number of trials missed, then POD is defined as

(16) $$\text{POD}=\frac{N_{D}}{N_{D}+N_{M}}\text{.}$$

POD represents the true positive rate of certain brain states. Two values were calculated based on POD: POD of MI tasks and POD of idle states (*relax* task). For POD of MI tasks, we only care if MI tasks could be discriminated from idle states. Whether MI tasks were classified correctly is another issue. For a practical BCI system, the POD of idle states is critical because false alarms during idle states may lead to unexpected action of the BCI system when the subjects are resting or idling.

(b) *Classification accuracy (CA)*

For a certain MI task A, considering all trials of task A, let *N*_*C*_ denote the number of trials correctly classified as task A, *N*_*W*_ the number of trials classified as other MI tasks, then CA is defined as

(17) $$\text{CA=}\frac{N_{C}}{N_{C}\;+N_{W}}\text{.}$$

According to this definition, the number of trials classified as idle states is not included in (17). It is easy to understand: failure of detection will not lead to execution of improper commands; only the average time for carrying out one command will be lengthened. From this point of view, the POD of MI tasks together with CA decides the speed of the synchronized BCI system. The mean CA value of both MI tasks was calculated as the average CA.

Referring to our proposed criterion for selecting thresholds, *k*_1_−*k*_4_ were decided by **P**_1_ and *P*_2_. Varying these two probabilities leads to changes of the performance measures defined above. Ideally, a BCI system with good performance is associated with CA, POD of MI tasks and idle states close to 100%. Therefore, **P**_1_ and *P*_2_ should be chosen carefully to make the real BCI system with a good performance.

To simplify this problem, we make both **P**_1_ and *P*_2_ equal to a certain value *P*. Table 2 listed the results calculated by increasing *P* from 0.6 to 1 in step of .05 for subject FL. The values of CA and POD_Idle_ are negative correlated with *P*, while POD_MI_ is positive correlated with *P*. The basic principle for choosing *P* value is to reduce false alarm during idle states (i.e., increase POD_Idle_) while keeping POD_MI_ and CA at an acceptable level. The optimal *P* value for subject FL is manually selected as 70% with high CA and POD_Idle_ as well as a relatively high POD_MI_ (see Table 2). In the same way, the optimal *P* values for the other two subjects are chosen as 80% (ZD) and 90% (ZYJ); the corresponding results are shown in Table 3. The data of all three subjects achieved nearly 100% CA for discriminating the two MI tasks, with average POD_MI_ about 70% and average POD_Idle_ above 90%.

## 4. CONCLUSIONS AND DISCUSSION

The most important characteristic of our algorithm was combining two two-class classifiers to construct a three-class classifier. We broke down the problem into two steps and solved them consecutively with parameters separately optimized in each step for its own purpose. The analysis of the final result validated this strategy.

**Figure 7:**
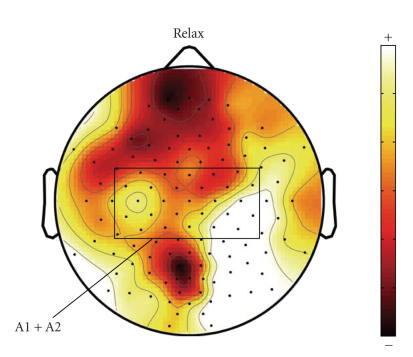
Averaged spatial mapping of *relax* (calculated in a same way as in Figure 1) in the testing set.

**Figure 8:**
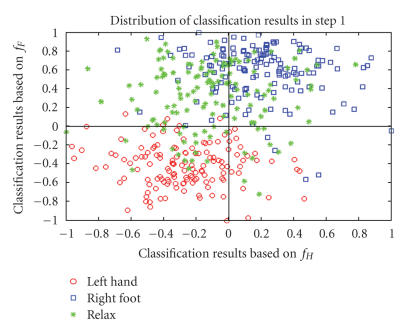
Distribution of classification results in Step 1.

The basic assumption was that during *relax* task there is no obvious ERD over somatosensory or motor cortex. This assumption is shown to be reasonable according to the final results. Figure 7 displays the averaged spatial mapping of *relax* (calculated in a same way as in Figure 1) in the testing set. There is no obvious ERD in region A1 and A2. Figure 8 shows the classification results of the samples in the testing set by these two classifiers and the true labels are given by different legends. Most samples of *relax* are located in the second quadrant, while *right-foot* and *left-hand* samples are in the first and third quadrants. This distribution is in accordance with the analysis inTable 1.

In Section 2.1 we listed three problems in dataset IVc, our algorithm addressed the problem of no training data for *relax*. The other two problems may lead to nonsignificant interference with the application of CSSD algorithm, which is essentially determined by the spatial patterns of different MI tasks.

The problems of shortened trial time and long-term nonstationarities seem to be not so critical here because the two MI tasks still can be discriminated well (see Figure 6). One possible reason is because this data set is from a very good subject (classification accuracy on training set is around 99%). For subjects with ordinary performance, the results might be worse. However, most subjects could achieve better performances after a certain period of training.

Another issue worth mentioning is the difference between *relax* and idle states. *Relax* might be slightly different from idle states, which are always referred to a quite long period with no MI going on rather than 1-second trials in these testing sessions. The brain states during *relax* trials in the testing sessions could be better described as “noncontrol” or “non-MI” states. In our algorithm, *relax* trials are only considered as brain states quite different from MI trials and no information were retrieved from these trials for designing the algorithm. From this point of view, we consider them as equal terms in this paper.

The traditional ways of idle-state detection mainly focus on developing powerful and robust algorithm mathematically. Our strategy aims at building a practical BCI system. In our opinion, how to integrate these methods in an effective way is also very important. Because the nature of idle states is quite different from those MI states, it is worth to set up an additional step with optimal parameters for separating these *relax* trials from the rest trials.

The proposed algorithm achieved satisfied results on our MI datasets. It shows the effectiveness of our algorithm for practical BCI systems. This result is also much better than dataset IVc of BCI competition III. The main reason might be due to the lengthened trial time, which is important for the subjects' performance.

The probabilities (**P**_1_ and *P*_2_), which decide the thresholds *k*_1_−*k*_4_, are crucial to the performance of our algorithm. For dataset IVc, we simply select 70% for both **P**_1_ and *P*_2_ based on our former experiences. These *P* values could be carefully chosen to make the performance better based on the three indexes (POD_MI_, POD_Idle_, CA) defined above. Decreasing *P* value will lead to higher POD_Idle_ but lower POD_MI_, which is the key factor for the speed of the synchronized BCI system. Also higher CA will be achieved because more ambiguous MI trials are labeled *relax*. Our current strategy is to insure a high POD_Idle_ (i.e., above 90%) first, and then make POD_MI_ and CA as high as possible. We have not established an automatic way to make a balance between POD_MI_ and POD_Idle_ yet. These results might be further improved by selecting optimal thresholds *k*_1_−*k*_4_ based on advanced statistical theories.

A BCI system that can distinguish patterns not included in training data is very attractive. Solving the problem of dataset IVc is a good step towards this target. The proposed algorithm is especially useful to reduce the false alarms in current BCI system based on MI when the subjects are not performing MI tasks. Although we perform offline analysis here, this algorithm could be easily moved to online system.

## Figures and Tables

**Table 1:** Ideal classification results of the three tasks.

Feature task	*f*_*F*_	*f*_*H*_	(*f*_*F*_ + *f*_*H*_)/2

*left hand*	−1 (ERD in A1)	−1 (no ERD in A2)	−1 (−1/−1)
*right foot*	+1 (no ERD in A1)	+1 (ERD in A2)	+1 (+1/+1)
*Relax*	+1 (no ERD in A1)	−1 (no ERD in A2)	0 (+1/−1)

“(*f*_*F*_ + *f*_*H*_)/2” represents the mean value of two outputs corresponding to *f*_*F*_ and *f*_*H*_ in the same row.

**Table 2:** Performance measures of subject FL corresponding to different *P* values.

*P* (*P*_1_&*P*_2_)	**POD**_MI_	**POD**_Idle_	**CA**

100%	100.0 ± 0.0%	0.0 ± 0.0%	89.0 ± 2.3%
95%	96.1 ± 1.8%	4.2 ± 1.2%	94.9 ± 1.8%
90%	90.0 ± 1.6%	61.2 ± 2.1%	96.8 ± 1.1%
85%	84.2 ± 2.3%	71.0 ± 3.2%	97.2 ± 2.5%
80%	74.1 ± 1.9%	81.4 ± 1.8%	96.6 ± 2.1%
75%	65.3 ± 2.2%	91.0 ± 1.6%	97.6 ± 1.4%
70%	62.7 ± 3.2%	95.5 ± 0.9%	98.9 ± 0.8%
65%	51.8 ± 2.0%	98.1 ± 2.2%	98.7 ± 1.0%
60%	45.1 ± 1.6%	99.6 ± 0.9%	99.3 ± 1.2%

**Table 3:** Performance measures of three subjects with the optimal *P* values.

**Subject**	**Optimal** *P*	**POD**_MI_	**POD**_Idle_	**CA**

ZYJ	90%	78.2 ± 1.7%	90.2 ± 1.3%	98.3 ± 1.2%
FL	70%	62.7 ± 3.2%	95.5 ± 0.9%	98.9 ± 0.8%
ZD	80%	61.2 ± 2.2%	96.1 ± 1.1%	99.4 ± 0.4%

## References

[1] Wolpaw JR, Birbaumer N, McFarland DJ, Pfurtscheller G, Vaughan TM (2002). Brain-computer interfaces for communication and control. *Clinical Neurophysiology*.

[2] Pfurtscheller G, Neuper C (2001). Motor imagery and direct brain-computer communication. *Proceedings of the IEEE*.

[3] Jeannerod M (1995). Mental imagery in the motor context. *Neuropsychologia*.

[4] http://ida.first.fraunhofer.de/projects/bci/competition_iii/desc_IVc.html.

[5] Pfurtscheller G, Lopes da Silva FH (1999). Event-related EEG/MEG synchronization and desynchronization: basic principles. *Clinical Neurophysiology*.

[6] Wang Y, Berg P, Scherg M (1999). Common spatial subspace decomposition applied to analysis of brain responses under multiple task conditions: a simulation study. *Clinical Neurophysiology*.

[7] Wang Y, Zhang Z, Li Y, Gao X, Gao S, Yang F (2004). BCI competition 2003—data set IV: an algorithm based on CSSD and FDA for classifying single-trial EEG. *IEEE Transactions on Biomedical Engineering*.

[8] Ramoser H, Müller-Gerking J, Pfurtscheller G (2000). Optimal spatial filtering of single trial EEG during imagined hand movement. *IEEE Transactions on Rehabilitation Engineering*.

[9] Garrett D, Peterson DA, Anderson CW, Thaut MH (2003). Comparison of linear, nonlinear, and feature selection methods for EEG signal classification. *IEEE Transactions on Neural Systems and Rehabilitation Engineering*.

[10] Breiman L (1996). Bagging predictors. *Machine Learning*.

[11] Dornhege G, Blankertz B, Curio G, Müller K-R (2004). Boosting bit rates in noninvasive EEG single-trial classifications by feature combination and multiclass paradigms. *IEEE Transactions on Biomedical Engineering*.

[12] Blankertz B, Müller K-R, Krusienski DJ (2006). The BCI competition III: validating alternative approaches to actual BCI problems. *IEEE Transactions on Neural Systems and Rehabilitation Engineering*.

